# Effect of Chewing Gum on Duration of Postoperative Ileus Following Laparotomy for Gastroduodenal Perforations: Protocol for a Randomized Controlled Trial

**DOI:** 10.29337/ijsp.188

**Published:** 2023-02-06

**Authors:** Joshua Muhumuza, Selamo Fabrice Molen, William Mauricio, Jorge Soria La O, Jethro Atumanyire, Nyenke Bassara Godefroy, Musa Abbas Waziri, Stephen Mbae Kithinji, Kiyaka Magugu Sonye, Mugisho Munyerenkana Leocadie, Franck Katembo Sikakulya, ByaMungu Pahari Kagenderezo, Musafiri Simba Lionel, Mumin Farah, Herman Lule

**Affiliations:** 1 Faculty of clinical medicine and dentistry, Department of Surgery, Kampala international University Western campus, Ishaka Bushenyi, Uganda; 2 Faculty of Medicine and dentistry, Kampala international University Western campus, Ishaka Bushenyi, Uganda; 3 State Specialist Hospital (HMB), Department of Surgery. Maiduguri, Borno State Nigeria; 4 Faculté de médecine, Université Catholique du Graben, Butembo, RDC; 5 Department of Clinical Medicine, Division of Clinical Neurosciences, University of Turku, FI-20014, Turku, Finland

**Keywords:** Chewing-gum, Ileus, Sham Feeding, RCT, Africa

## Abstract

**Background:**

Prolonged post-operative ileus is associated with increased risk of other complications, length of hospital stays and health care related costs. Chewing gum has been shown to reduce duration of ileus in many elective surgeries, but there is a paucity of randomised controlled trials (RCTs) on its effect on duration of ileus among patients undergoing emergency surgery, specifically patients with peritonitis. The aim of this study is to determine the effect of chewing gum on duration of postoperative ileus following laparotomy for gastroduodenal perforations.

**Methods:**

This will be a randomised controlled trial done in 3 hospitals. Fifty-two patients will be randomised to 2 groups. Group A will receive chewing gum in addition to routine care, whereas group B will receive routine care only. The duration of post-operative ileus in the two groups which is the primary outcome, will be compared using the independent samples t-test in SPSS version 22. The length of hospital stay, in-hospital morbidity and mortality will be the secondary outcomes. This trial has been approved by Kampala International University research and Ethics committee (Ref No. **KIU-2021-60**) and Uganda national council of science and technology (Ref No. **HS1665ES**). Retrospective registration with the research registry has also been done (UIN: researchregistry8565).

**Highlights:**

- Prolonged post-operative ileus significantly contributes to adverse surgical outcomes
- Chewing gum has been shown to reduce duration of ileus in many elective surgeries
- There is paucity of RCTs on role of chewing gum following surgery for peritonitis

## Introduction

Post-operative ileus (POI) occurs when gut motility briefly stops following surgery with no identifiable mechanical cause [1]. Prolonged postoperative ileus (PPOI) is the form that does not resolve by the 4^th^ post-operative day [2]. This form has been associated with prolonged admission time, high costs of health care and increased morbidity [3]. A study done to evaluate effect of POI in patients undergoing colectomy showed that the cost of prolonged POI was equivalent to the cost of re-admission with another adverse complication [4].

Over the years, patients who develop PPOI have been treated by passing a nasogastric tube (NGT), stopping oral intake and correcting electrolyte imbalances [3]. The currently available data shows that placement of a nasogastric tube does not prevent POI and may even increase the risk of other complications [5]. For this reason, the current recommendation is to avoid routine NGT insertion and to ensure early removal for those in whom it is deemed necessary [6]. Multiple preventive and treatment measures for POI have been studied over the years but the currently recommended measure is adherence to enhanced recovery after surgery (ERAS) protocols [5].

Chewing gum is reported to improve gut motility by activating the cephalic-vagal pathway that results in stimulation of gut myoelectric activity and intestinal movement [7]. This vagal stimulation is also thought to have an anti-inflammatory effect [8]. The first study that used chewing gum for POI resolution was done in 2002 among colon cancer patients [9]. This study reported reduced time to passage of stool, flatus and length of hospital stay [9]. Over the years more studies have been done in different study populations including laparoscopic, open, elective, emergency, colorectal, gynecological and many others with varying reports [1], [10,12].

The composition of chewing gum is chicle, a naturally occurring latex product [10]. Both sugar free and sugared chewing gum have been used [13,14]. The cost of PK, a sugared type of chewing gum (CG) is 250 Uganda shillings (0,065$) for a 5.6 g pack. The Ingredients of PK include sugar, gum base, glucose syrup, modified starch, emulsifier, soybean, lecithin, flavorings, glycerol, and an antioxidant (PK pack manufacture’s claim). In relation to the down sides of chewing gum, it cannot be given to patients that are not fully awake [15]. Also a sugared type of chewing gum has been reported to increase risk of dental curies [16], though rinsing and brushing regularly have been reported to mitigate this side effect [16,17].

Chewing gum has been studied in different settings of surgery including colorectal, appendectomy, Gynecological operations, caesarean section and some studies have involved different abdominal surgeries including both electives and emergencies [7,12,18,19,20]. One meta-analysis published in 2017 showed that in most of the studies done, patients were told to chew gum three to four times a day [21]. The duration for which patients chewed has been ranging from five to sixty munities as shown in one meta-analysis published in 2016 [22]. The quantity of gum chewed at each time has not been standard but statements like one stick, one pellet or two pellets have been used and in most studies patients usually start chewing within 24 hours after surgery when patient is fully awake [10,23].

### Rationale

There is paucity of data about treatment of POI and most of the medicines studied have had unsatisfactory outcomes [8]. For this reason most of the emphasis is currently on preventive management [21]. ERAS protocols have been proven to reduce POI duration in elective surgeries but data about such protocols in emergency surgeries is lacking [24]. The elements of ERAS recommended for reduction of POI in emergency surgeries, have been based on law risk patients [5,24], yet most of the patients in resource limited countries present late with a high risk of peritonitis and morbidity [25]. More so, some doctors avoid early feeding in fear of the complications since up to 20% of the patients do not tolerate it [11]. The minimally invasive surgery, a major component of ERAS is still a dream to most of the hospitals in resource limited countries. Most of the studies on chewing gum in POI were done in elective operations mainly colorectal. The mixed studies that included emergency operations, excluded patients that had gut perforations or peritonitis [7]. This study is necessary to understand if chewing gum can be effective in reducing postoperative ileus duration in patients with peritonitis.

### Study objectives

#### Main objective

The main objective in this trial is to determine the effect of chewing gum on duration of postoperative ileus following laparotomy for gastroduodenal perforations in a resource limited setting.

#### Specific objectives

- To compare the time taken for post-operative ileus to resolve in the group of patients chewing gum versus the controls.
- To compare the duration of hospital-stay in the group of patients chewing gum versus the controls.
- To compare in-hospital morbidity and mortality in the group of patients chewing gum versus the controls.

### Hypotheses

The null hypothesis of this study is that there is no difference in the duration of POI when gum is chewed compared to when it is not.

## Methods

### Study design

This will be a multicenter prospective randomized controlled study with a parallel superiority design conducted in the surgical departments of Kampala International University Teaching Hospital (KIU-TH), Hoima regional referral Hospital (HRRH) and Fort portal regional referral Hospital (FRRH) in western Uganda. This work has been reported in line with the consort criteria [26].

### Study setting

HRRH has a bed capacity of about 600 with 3 general surgeons and 2 senior house officers. FRRH has a bed capacity of about 333 with 2 general surgeons and 3 senior house officers. KIU-TH has a bed capacity of about 700 with 6 general surgeons and 30 senior house officers. The surgery departments in the 3 study centers do both emergency and elective surgeries including the repair of gastric and duodenal perforations. The senior house officers do the operations under supervision of the general surgeons.

### Study population

All adult patients admitted to the surgical wards with a gastric or duodenal perforation during the study period will be targeted.

### Eligibility criteria

#### Inclusion Criteria

All patients with confirmed gastric or duodenal perforation, between the age of 18 and 65 will be included. The upper age limit will be used because increasing age increases the risk of PPOI [10] yet we want the groups to be comparable.

#### Exclusion Criteria

- patients with documented allergies to the contents of chewing gum
- Patients with uncontrolled diabetes mellitus disease at the time of surgery since we are using a sugared type of chewing gum that may worsen the glycemic status.
- Patients who will be unconscious or unable to chew gum after 24 hours post-surgery
- Patients with traumatic perforations will be excluded.

### Sample size estimation

Using the formula; N = 2\; \times \,{({\textstyle{{{Z_{1 - \alpha /2}}\; + {Z_{1 - \beta }}} \over \delta }})^2} \times {S^2} for determining sample size in randomized clinical studies with a statistical superiority design [27], and findings from a study that evaluated effect of chewing gum following pancreatic-duodenal surgery in Sweden [28], 46 participants per group will be required. On adjusting the sample size to the finite population and adding 10% to improve internal validity, the required sample size will be 52.

### Sampling technique, randomization, concealment allocation and blinding

Permuted balanced block randomization will be conducted by the study supervisor to generate a random sequence of numbers to represent the two groups in an equal allocation ratio of 1:1. The random numbers will be labeled on concealed envelopes. The sealed envelopes will contain a folded opaque paper with letter A or B representing (chewing plus routine care) and (non-chewing plus routine care) groups respectively so that its not possible to deduce the assignment group based on random sequence numbers by the allocating team (research assistants). In addition, the envelope will have a similar stamp on the seal to be able to deduce possibility of breach of protocol (premature opening of the envelope). At this time both consent for surgery, information about the study and or consent to participate in the study will be obtained in writing at the emergency department. This is to avoid bias of recruiting participants after knowledge of intra-operative findings and to enable pre-operative care standardization between groups. The allocating team (trained research assistants) will give the envelope to the eligible participant 2 hours prior to surgery when a diagnosis of gut perforation is made from the radiological findings. The envelope will remain sealed until the patient enters the operating room and the participant will be unblinded just before induction of anesthesia where an instruction will be issued regarding chewing or not chewing any gum post-operatively. At this point an additional number either H, F or K will be added to the sequence number on the envelope to form participant identification number (PIN) to represent HRRH, FRRH, KIUI-TH for tracking records of recruitment and representation from each hospital. The outcome assessors (attending physicians) will be blinded throughout about the ongoing study since chewing gum is not known to cause adverse effects that would warrant un-blinding. The recruiting team will be totally different from the outcome assessors.

### Participant recruitment and study procedure

Patients admitted in emergency department (ED) with suspected gastric or duodenal perforation based on clinical and radiological assessment will be evaluated for eligibility and considered as possible candidates for the study.

Routine pre-operative management for GDP will be done for all patients including; taking vital signs like; Blood pressure (BP), pulse rate (PR) respiratory rate (RR), oxygen saturation (SPO2) and temperature plus continuous monitoring, passing Foley’s urethral catheter and monitoring urine output, passing two large bore cannulas, fluid resuscitation with IV Normal saline till urine output is >0.5 mls/h, nasogastric tube insertion, nil by mouth, intravenous (IV) ceftriaxone 2 grams (g), IV metronidazole 500 milligrams (mg), IV Paracetamol 1g, IV omeprazole 40 mg, doing complete blood count (CBC), doing blood grouping and cross match, serum electrolytes, and obtaining informed consent for emergency laparotomy.

After resuscitation, the patients will be taken for laparotomy under intubation general anesthesia. Patient’s particulars will be recorded in the data collection form including participant identification number (PIN), age, address, presenting symptoms and duration of symptoms among others as shown in appendix I. The type of perforation surgery will be recorded.

**Figure 1:**
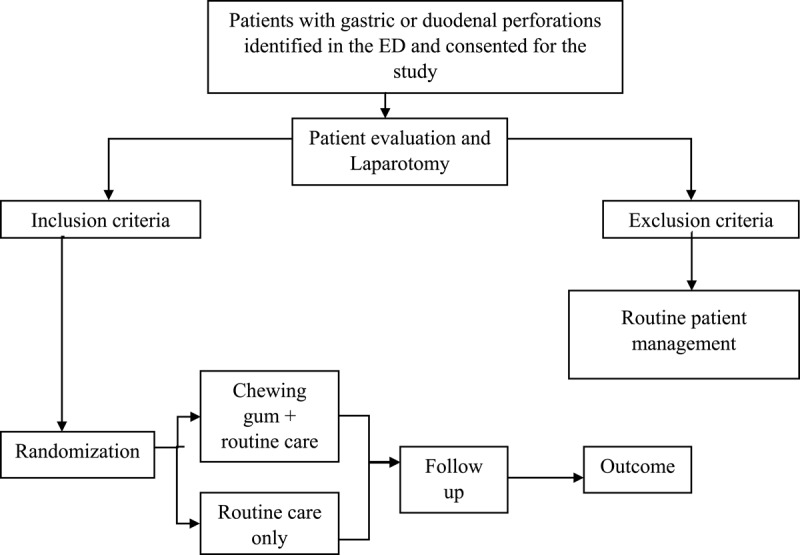
Flow chart for sampling and data collection procedure.

Post operatively patients in both groups will receive all the routine post-operative care. This will include IV Normal saline and 5% dextrose in the ratio of 2:1, which will be prescribed for the first 24 hours with the total volume determined by the 4:2:1 rule for calculating maintenance fluids. The fluid prescriptions that follow, will be according to fluid balance chart. Other post-operative instructions will include; IV ceftriaxone 2g once a day (OD) for 5 days, IV metronidazole 500mg 8 hourly for 5 days, IV Omeprazole 40mg OD till oral feeding is initiated, IV Paracetamol 1g 8 hourly till oral feeding is initiated, Oral warm water will be started on first POD, early removal of NGT will be done, ambulation will be encouraged from first POD [14,25]. In addition to the above, patients in group A will receive chewing gum and below is a clear instruction on how they will use it. Figure 1 shows a flow chart summarizing the study procedure.

Any breach or failure to adhere to pre or post-operative protocol will lead to termination of the individual participants from the RCT. Adherence to the trial protocols will be checked for in the patients’ treatment record charts from the time of admission to the end of the follow-up period.

### Chewing gum administration

Green PK brand of chewing gum will be used in this study since a pilot survey done before data collection showed that majority of the people preferred the green flavor (*appendix II, unpublished data*). We will use one flavor to minimize bias. The patients in Group A will be given green flavored PK and will start chewing after regaining consciousness. The patients will chew 2 pellets of PK till they are tasteless three times in a day (morning, afternoon, and evening). After chewing, the patients will be encouraged to rinse the mouth with clean water. The patients will stop chewing gum when POI resolves. Resolution of POI will be defined as passage of stool or flatus or both in addition to full filling the criteria defined in the 6 domains described by the Tripartite gastrointestinal recovery post-operative ileus group such as absence of nausea and vomiting, having no need of nasogastric intubation, absence of abdominal pain and distension, and ability to tolerate oral feeds [29]. Each patient will be offered a watch, notebook, and pen and with the help of the caretaker, record every day’s events and the time they occur. The events recorded will include; time when gum is chewed, flatus passed, stool passed, vomited or hunger felt. The outcome assessors will do clinical evaluation 6 hourly and information will be recorded in the data collection form (appendix I).

### Study variables

Primary outcome variable will be time taken from end of surgery to resolution of ileus.

Secondary outcome variables will be duration of hospital stay in days (counted from time of admission to time of discharge), in-hospital morbidity and mortality.

Morbidity will be defined as any deviation from the postoperative course that is not inherent in the procedure and does not comprise a failure of cure and will be reported in accordance with the Clavien Dindo classification 2004 [30]. Grade I: Any complication that does not require additional pharmacological, radiological or surgical intervention, II: Requires pharmacological treatment but not radiological or surgical intervention eg blood transfusion, III: Requires radiological or surgical re-intervention eg endoscopy and IV: Life threatening complications eg atleast one organ failure requiring ICU admission.

### Validity of the data collection tools

A Pilot study was performed to pre-test the questionnaire and confirm its effectiveness, by using a content validity index. Two surgeons in Hoima were given the questionnaire and content validation form was prepared. The surgeons reviewed both the domain and the item and assigned scores. The content validity index was calculated. A content validity index of at least 0.80 was considered acceptable as recommended by Davis [31] after making necessary adjustments.

### Reliability of the data collection tools

Reliability analysis was conducted before starting data collection. A Cronbach’s coefficient alpha of ≥ 0.70 was considered for a questionnaire to be used to collect the data. Such a Cronbach’s coefficient alpha implies the questionnaire elements are reliable and reproducible.

### Data processing and analysis

The intention to treat model will be used during analysis to minimize bias. Summarized data will be analyzed using Statistical Package for the Social Sciences (SPSS Inc., Chicago, USA, version 22.0 for Windows). Continuous variables will be presented as the mean ± standard deviation. Categorical variables will be expressed as frequencies.

To compare the time taken for post-operative ileus to resolve and length of hospital stay, normality of distribution will be assessed using the Shapiro-Wilks test. If the data are found to be normally distributed, then equal variance for the two groups will be determined using Levene’s test hence two sample t-test will be computed assuming equal or unequal variance as appropriate. If the data are not normally distributed in both groups, the nonparametric Wilcoxon Rank sum (Mann-whitney U) test will be used to compare the difference in means. To compare the in-hospital morbidity and mortality, the chi squared test will be used. All analyses will be performed at 95% confidence level and a p value ≤0.05 will be considered significant.

### Data management

The data collection forms will have patient PIN code instead of names and will only be accessible to investigators. Hard copy records will be kept in a locked cabinet until 6 months following completion of the study and thereafter will be destroyed. Electronic records will be kept in a password protected file on the investigator’s password protected computer, and on completion of the study, they will be saved on a Digital Versatile Disc (DVD) that will be submitted to KIU registry and to a peer reviewed journal’s permanent data repository link for accessibility and reusability by other researchers.

### Ethical considerations

The clinical trial was assessed and approved by the Research and Ethics Committee of Kampala International University (KIU-REC reference number **KIU-2021-60**). After approval, the study was registered by Uganda National Council for Science and Technology (UNCST reference number **HS1665ES**). This protocol has been retrospectively registered with the research registry UIN: **researchregistry8565** hyperlink: https://www.researchregistry.com/browse-the-registry#home/. Using the standard KIU-REC consent form, written informed consent will be obtained from each of the study participants. The consent form has been translated into local languages. A consent form for Surgery conforming to the world health organization (WHO) surgery consent content will be used to obtain informed consent before surgery. All the patients full filling the inclusion criteria in the study population will have the same chance to be in the study. There will be no preferential treatment based on race, color, social status, economic status or any other. In terms of risks, studies have shown that chewing gum after surgery is safe, thus the study poses not more than minimal risk. There will be no personal reward or incentive for participating in this study, however the money to pay for investigations required for the study (CBC and electrolytes) will be covered by the investigators. To minimize spread of Covid-19 infection, regular hand washing or alcohol hand rub, social distancing, use of appropriately worn face mask, screening for temperature and other prevention strategies as recommended by the Uganda ministry of health from time to time will be adhered to. The Uganda National Guidelines for currying out Research in COVID- 19 pandemic (UNCST, 2020) will be followed.

### Interim analysis

The research and ethics committee considered this study “minimal risk” that would not warranty interim analysis. The committee in principle performs its independent auditing of the study and can terminate, ask modification, or recommend continuation of the study at any time without prior notification.

### Termination from the study

Individual participants will be terminated from the study if they do not adhere to protocol for instance cross-over to cause contamination, breach allocation or are lost to follow-up.

## Discussion

Previous researchers have not focused on acute peritonitis due to gastroduodenal perforations when assessing effect of chewing gum on ileus which this study is set to address. In their findings so far, there is compelling evidence to suggest that chewing gum plays a role in resolution of ileus [9,12], although controversial findings have been reported as well [28]. Our study is timely to shade more light in the context of low resource settings that are often under-represented in research publications.

Readily available and affordable chewing gum could offer benefit of reducing prolonged post-operative ileus and result in an added advantage of being discharged through a short-hospital-stay track. This protocol seeks to generate level one evidence regarding the role of chewing gum in resolution of ileus among patients with peritonitis due to gastro-duodenal perforations.

The generalizability of our study is limited by relatively smaller sample size and excluding patients with comorbidities but we hope our findings could be used to benchmark larger future multi center randomized controlled trials. More so, in this study, the participants will be un-blinded early since any placebo in this case would also count as sham feeding. We will only enroll patients with peritonitis due to gastroduodenal perforations which may not have a high bacterial burden compared to other gastrointestinal perforations thus generalizability of results will be limited in this context.

## Data accessibility statement

The data generated from this protocol will be publicly available through a peer reviewed journals’ data repository link. The data collection materials are available through the corresponding author.

## Additional Files

The additional files for this article can be found as follows:

10.29337/ijsp.188.s1 Appendix I. Data collection form.

10.29337/ijsp.188.s2 Appendix II. Results of pilot survey on preferred type of chewing gum.

## Research registration

This protocol has been retrospectively registered with the research registry UIN: **researchregistry8565** hyperlink: https://www.researchregistry.com/browse-the-registry#home/.

## Trial updates

The first participant was recruited on 01^st^ April 2022.
