# Supplementary material for: Effect of Chewing Gum on Duration of Postoperative Ileus Following Laparotomy for Gastroduodenal Perforations: Protocol for a Randomized Controlled Trial
Source: Int J Surg Protoc. 2023 Feb 6;27(1):9–17. doi: 10.29337/ijsp.188 (PMC9912851; doi:10.29337/ijsp.188)
Supplement: Appendix I. — Data collection form. [file ijsp-27-1-188-s1.pdf]

Appendix I: Data collection form

**PRE-OPERATIVE**					
**Patient particulars**					
PIN		Age		Sex	
Village		S/county		District	
Tribe		Contact		Occupation	
**Clinical History**					
Date of admission		Time of admission			
Symptoms	**Yes**	**No**	**Duration**		
Abdominal pain					
Vomiting					
Nausea					
Fever					
Loss of appetite					
History of PUD					
Smoking history	Never	Stopped	when	Smokes	since
Use of NSAIDS	**< 1 week ago**	**> 1 week ago**	**Never**		
Alcohol intake	Never	Stopped	When	Takes	since
previous operations	**Yes**				
	**No**				
Other comorbidities	HIV	**Yes**	**No**		
	HTN	**Yes**	**No**		
	COPD	**Yes**	**No**		
	Others				
**Examination findings on admission**					
Weight		Height		BMI	
Temperature		RR		SPO2	
BP1		BP2		PR	

Distension		Tenderness		R/Tenderness	
Bowel sounds	**Present**	**Absent**			
Urine out Put	**Nil**	**<0.5/kg/hr.**	**> 0.5/kg/hr.**		
**INTRAOPERATIVE FINDINGS**					
Medication used in anesthesia					
Operation date					
Starting time		Ending time			
Duration		Site of perforation			
Size of perforation		Volume of p/fluid			
Type of fluid in peritoneum	**Flank pus**	**No flank pus**			
Other intra operative findings					
Type of operation done					
**POST OPERATION**					

Serum Electrolytes	Date							
	K							
	Na							
	Cl							
	Use or no use							
NGT	Date							
	Volume							
	Time removed							
Oral sips	Date							
	Time started							
	Number of times							
Complete blood count (CBC)	Date							
	Hemoglobin (HB							
	Leucocytes							
	Platelets							
Transfusion	Date							

	Blood component							
	Number of units							
Ambulation	Date							
	Time started							
	Number of times							
Analgesia	Date							
	Drug							
	Dose							
	No of times							
Antibiotics	Date							
	Drug							
	Dose							
	Number of times							
PPI	Date							
	Drug							
	Dose							
	Number of times							
Chewing gum	Date							
	Time started							
	Number of times							
	Chewing duration							
	Number of pellets							
	Challenge with CG							
Re operation	Date							
	Indication							
	Operation							
Flatus	Date							
	First time passed							
	No of times							

Stool	Date							
	First time passed							
	No of times							
Bowel sounds	Date							
	Time first heard							
	Number per min							
First feeling of hunger	Date							
	time							
Vomiting	Date							
	Number of times							
	Color							
	Last vomiting							
Nausea	Date							
	Time							
	Time stopped							
Complications like; Sepsis, Surgical site infection, Pneumonia, Fistula, Burst abdomen and others	Date diagnosed							
	Time diagnosed							
	Complication							
Mortality status								

**DISCHARGE**		
Date	Time	Status
