# Supplementary material for: Effect of Chewing Gum on Duration of Postoperative Ileus Following Laparotomy for Gastroduodenal Perforations: Protocol for a Randomized Controlled Trial
Source: Int J Surg Protoc. 2023 Feb 6;27(1):9–17. doi: 10.29337/ijsp.188 (PMC9912851; doi:10.29337/ijsp.188)
Supplement: Appendix II. — Results of pilot survey on preferred type of chewing gum. [file ijsp-27-1-188-s2.pdf]

Appendix II

Results of pilot survey on preferred type of chewing gum.

20 patients admitted in the surgery department of Hoima regional referral hospital were presented with 4 flavors of PK chewing gum (yellow, green, blue and red) and asked to state which their first and second choices would be. The responses were documented in a table and the preferences computed. It was observed that the first choice of the majority was green (70%). Of the remaining 30% that had another flavor as their first choice, 25% mentioned the green flavor as their second choice. Only 5% of the respondents did not mention the green flavor as either 1st or 2nd choice. This implied that 95% of the respondents could take the green flavor of PK chewing gum.
